# Neurodevelopment and psychosocial adjustment in children with early surgical ventricular septal defect repair: exploring the influence of surgery-related variables across childhood and adolescence

**DOI:** 10.1186/s13019-026-03897-1

**Published:** 2026-03-31

**Authors:** Jennifer Gerlach, Jonas M. Hemetsberger, Ariawan Purbojo, Robert A. Cesnjevar, Oliver Kratz, Anna Eichler

**Affiliations:** 1 Department of Child and Adolescent Mental Health, University Hospital Erlangen, Friedrich- Alexander-Universität Erlangen-Nürnberg (FAU), Erlangen, 91054 Germany; 2 Department of Pediatric Cardiac Surgery, University Hospital Erlangen, Friedrich-Alexander-Universität Erlangen-Nürnberg (FAU), Erlangen, 91054 Germany; 3 Department of Pediatric Cardiovascular Surgery, Pediatric Heart Center, University Children’s Hospital Zürich, Zürich, 8032 Switzerland

**Keywords:** Congenital heart disease, Ventricular septal defect, Pediatric cardiac surgery, Neurodevelopment, Psychosocial adjustment, Longitudinal study

## Abstract

**Background:**

This study investigates the effects of surgery-related variables on child neurodevelopment, internalizing symptoms, health-related quality of life (HRQoL), and maternal psychopathology following early child surgical Ventricular Septal Defect (VSD) repair.

**Methods:**

24 children (54.2% females) who underwent VSD surgery (age at surgery: Range 1.38 to 33.18 months) and their mothers were examined from child primary school-age (T1; *M* = 7.3 years, *SD* = 0.99) to early adolescence (T2; *M* = 12.4 years, *SD* = 0.93). Surgery-related variables (age at surgery, duration of surgery, cardiopulmonary bypass time, post-surgical hospital stay) were retrospectively reviewed, surgical scar characteristics were measured at T2. Child neurodevelopment, mother-reported internalizing symptoms and HRQoL were assessed at both time points, with adolescents also rating their internalizing symptoms and HRQoL at T2. Data were analyzed using repeated measures analyses of covariance (T1-T2).

**Results:**

An older age at surgery was linked to lower child long-term HRQoL. Time-based surgery-related variables (duration of surgery, cardiopulmonary bypass time) were associated with higher HRQoL at T1, while a longer duration of surgery affected maternal psychopathology at T2. A prolonged post-surgical hospital stay was negatively related to child neurodevelopment, and negatively predicted internalizing symptoms and child-rated HRQoL at T2. Longer surgical scars were correlated with higher child anxiety at T1 and lower long-term HRQoL, while wider scars were associated with increased depressive symptoms and lower HRQoL over time, and higher maternal psychopathology at T1.

**Conclusions:**

In sum, surgery-related variables primarily influenced child long-term psychosocial adjustment, with less impact on neurodevelopment. Postoperative variables have demonstrated greater relevance compared to time-based surgery-related variables. Effective management during all surgical phases is therefore crucial for both somatic and psychosocial recovery.

Out of every 1000 children, 8–10 are born with a congenital heart disease (CHD) [1, 2]. Of these, approximately 37% are isolated ventricular septal defects (VSD) [3]. Although the rate of spontaneous VSD closure is highly variable [4], VSDs require surgical correction in 20% of children [5]. If possible, the standard of care is to perform surgery before the age of 1 year and to try to limit the postoperative intensive care unit stay to 1 day in the absence of surgical complications [6].

Advances in surgery and medical care have greatly improved survival rates, allowing patients with CHD to lead a lifestyle associated with improved somatic health. However, research has revealed several impairments in neurodevelopment and psychosocial adjustment in CHD-affected individuals [7, 8]. Most long-term studies reporting on the neurodevelopment of patients with CHD have shown that affected children are at high risk of developmental delay [9, 10]. The severity of the impairment seems to increase with the complexity of the CHD [11]. However, studies focusing on the understanding and improving of psychosocial adjustment and well-being of patients with CHD are still rare [12]. Moreover, the existing literature on the early socio-emotional development and mental health of children with CHD revealed a paucity of conclusive results regarding internalizing (emotional) and externalizing (behavioral) symptoms in infancy and childhood. Specifically, the literature indicates conflicting findings; some studies have reported higher prevalence among children with CHD compared to non-affected controls, while other studies have not yielded similar findings [8, 13].

Importantly, CHD does not only affect the child but places significant psychological and emotional demands on the entire family system. In particular, mothers appear to be at elevated risk for mental health problems following their child’s pediatric cardiac surgery [14]. Research has demonstrated a strong association between maternal mental health and the psychological adjustment of children after early cardiac intervention [15–18]. These findings highlight the importance of incorporating parental mental health - especially that of mothers - into investigations of long-term psychosocial outcomes in children with CHD [14].

In addition to the overall negative effect of CHD on the child and its entire family, some studies discuss (long-term) effects of characteristics of the surgery itself. Many studies showed an adverse effect of surgery-related variables on children’s (long-term) quality of life [19–21]. Recent research estimates that surgery-related, biomedical variables explain between 24 and 29% of variance in the prediction of children’s total quality of life after CHD surgery [22]. Other studies focusing on severe CHDs reported that a longer stay in the intensive care unit or a longer post-surgical hospital stay as well as an older age at surgery were associated with more cognitive impairment [23] and more internalizing and externalizing symptoms [24]. Hövels-Gürich and colleagues [25] investigated the effects of surgery-related factors in a sample of patients with VSD and reported that a longer duration of cardiopulmonary bypass predicted higher attentional deficits at primary school-age. In addition, some studies have indicated negative effects of surgical scarring on children’s mental health, including the possibility of a reduction in self-esteem, confidence and positive body image [25–27].

However, studies focusing on the effects of surgery-related variables yielded conflicting results regarding the identification of influential factors [28], and the aforementioned studies frequently encompass a heterogeneous sample of diverse forms of CHD, exhibiting varying degrees of severity. Compared with more severe CHD, an early surgical correction of an isolated VSD is associated with positive somatic outcomes. Due the positive somatic prognosis after surgical VSD correction, patients with isolated VSD are less likely to be followed up, leaving a risk of overlooking secondary impairments [15]. Therefore, the present study examines the long-term neurodevelopment, psychosocial adjustment and well-being of a sample of children after surgical VSD repair (surgery before the age of 3 years) from primary school-age until early adolescence, taking into account the potential effects of surgery-related variables. In our previous study, in particular, a younger age at surgery as well as a short post-surgical hospital stay were predictive of higher child well-being at primary school-age. Furthermore, a longer post-surgical hospital stay was associated with lower IQ-scores (neurodevelopment) [15]. Therefore, we aimed to follow-up children’s neurodevelopment, internalizing symptoms (anxiety and depression) and HRQoL in adolescence testing the effects of surgery-related variables (age at surgery, duration of surgery, cardiopulmonary bypass time, post-surgical hospital stay, length and width of the surgical scar) on child development. As shown in previous studies, we expected a higher age at surgery, a longer duration of surgery or cardiopulmonary bypass time, a longer post-surgical hospital stay and a noticeable scar would be positively related to children’s anxiety and depression levels and negatively associated to their neurodevelopment and HRQoL [15, 24, 25]. As the mother plays a crucial role in the long-term psychosocial adjustment of the VSD-affected child [14–16], the effects of VSD surgery characteristics on maternal psychopathology were also explored.

## Method

### Study design

**Fig. 1:**
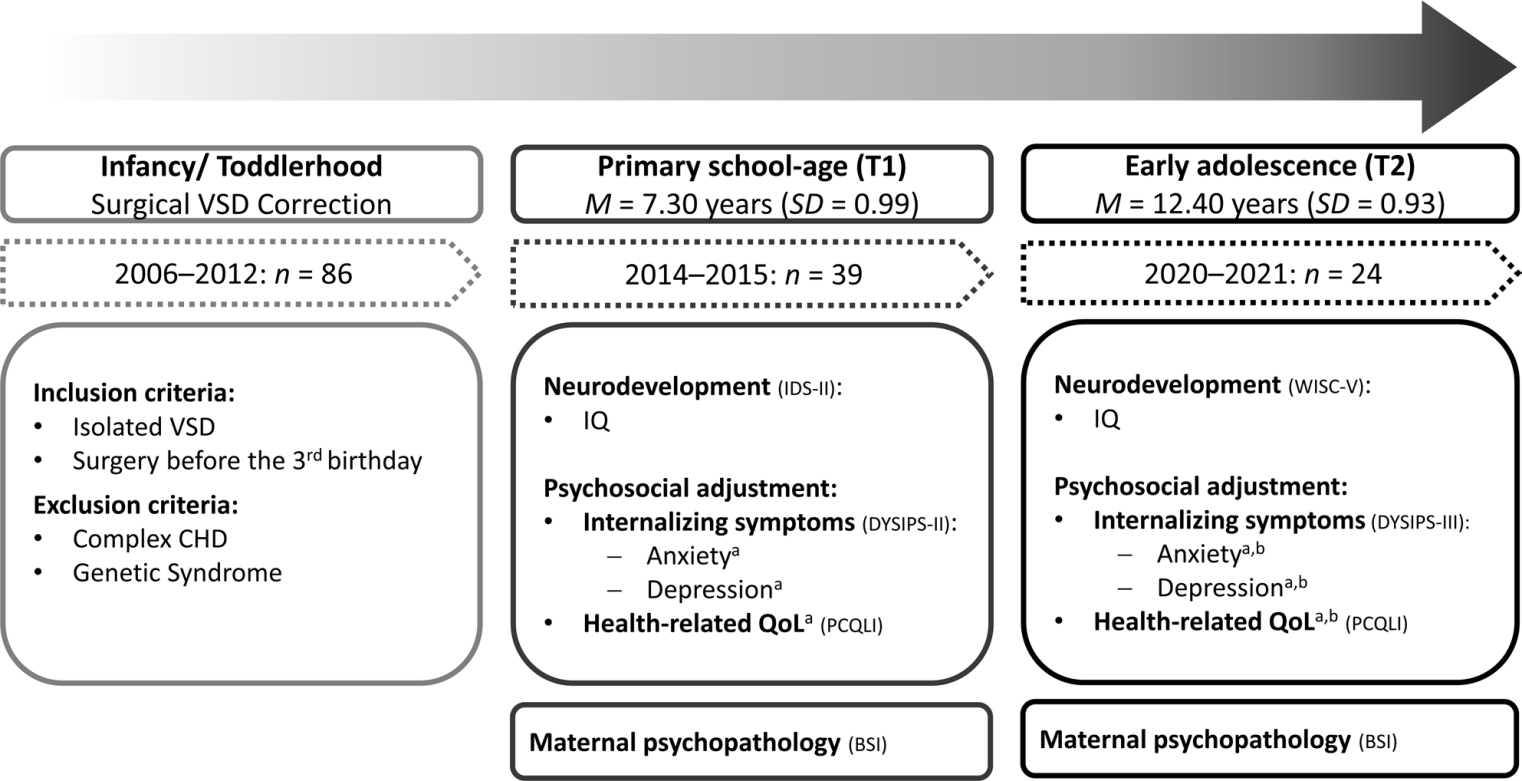
Study design. ^a^mother-report,^b^child self-report. IQ: T1, Intelligence and Development Scales, IDS-III [30]; T2, Wechsler Intelligence Scale for Children – Fifth edition, WISC-V [31]; child internalizing symptoms: T1, DYSIPS-II [32], T2, DYSIPS-III [33]; Health-related quality of life (HRQoL): PCQLI [34]; Maternal psychopathology: BSI [35]

This study is part of a long-term project investigating the development of children who underwent surgery for VSD under 3 years of age. During March 2006 to March 2012, a total of 86 children underwent surgical VSD correction at the Department of Pediatric Cardiac Surgery at the University Hospital Erlangen in Germany. In 2015, the Department of Child and Adolescent Mental Health invited families to participate in the study, yielding in 39 families that were enrolled in the first time of measurement (T1) [15]. From 2019 to 2021, a second time of measurement (T2) was performed when children were in early adolescence, and 24 families re-participated. Eight families were not interested in participating in the second time of measurement, six families had moved to an unknown address, and one family had moved to another country. Dropout analyses revealed no significant differences in families participating at T2 compared to those who dropped out in surgery-related variables, families’ socioeconomic status, and other variables of interest including child neurodevelopment, internalizing symptoms, HRQoL, and maternal psychopathology. See Fig. 1 for an overview of the study design.

The study was approved by the Local Ethics Committee of the Faculty of Medicine at the Friedrich-Alexander-Universität Erlangen Nürnberg (T1: 4596, 2 April 2014; T2: 353_18B, 12 April 2019), and all assessments were performed in accordance with the Declaration of Helsinki. The study is registered in the German Register for Clinical Trials (DRKS00024730). All mothers gave their written informed consent for study participation and publication of results, and all children gave their informed assent.

### Sample

At T1, children’s age ranged from 6 to 10 years (*M* = 7.30, *SD* = 0.99), in adolescence (T2), children were between 10 and 14 years old (*M* = 12.40, *SD* = 0.93). The gender ratio was balanced with 54.2% girls. At T2, mothers were between 30 and 50 years old (*M* = 40.69, *SD* = 8.03). Families’ monthly net income varied between 2000€ to 5000€, with 37.5% having 2000€ to 3000€ per month, 16.7% having 3000€ to 4000€, 16.7% having 4000€ to 5000€, and 12.4% having 4000€ to 5000€ per month; 16.7% did not provide any income information. Table 1 provides an overview of children’s cardiovascular problems in addition to the VSD and the complications that occurred after the surgical VSD repair.

**Table 1:** Children’s cardiovascular problems (in addition to VSD) and complication after surgical VSD correction

	Frequency (%)^1^	Occurrence at the age of	currently present
*Other cardiovascular disease (in addition to VSD)*			
Cardiac arrhythmia	1	0d	yes
Cardiac syncope	1	3y	yes
Dilated cardiomyopathy	1	0y	yes
Right aortic arch	1	0y	yes
*Complications after surgical VSD correction*		Duration after surgical VSD correction	
Cardiac arrhythmia	3		
AV block	1	0d	yes
Left bundle branch block	1	0d	yes
Inflammation of the scar	1	14d	no
Fluid retention (need for punctuation)	1	2d	no
Need for cardiac pacemaker	1	0y	yes
Medication			
Beta blockers	0	-	-
ACE inhibitors	0	-	-

^1^Multiple answers possible. *N* = 24; d = days, y = years

## Measures

### VSD surgery-related variables

The following data were collected from medical records: Age at surgery (months), duration of surgery (min), cardiopulmonary bypass time (min), and post-surgical hospital stay (number of nights). Characteristics of the surgical scar were established using a measuring tape. In early adolescence (T2), both the length and width of the scar (cm) were assessed. Children’s current height was significantly associated with the length of the scar (*r* =.51, *p* =.012), so that both length and width of the scar were relativized to the child’s current height. Length and width of the scar were positively correlated by trend (*r* =.39, *p* =.067).

### Child neurodevelopment

Children’s neurodevelopmental status was measured at primary school-age (T1) using the German versions of the Intelligence and Development Scales (IDS) [29], and in adolescence (T2) using the Wechsler Intelligence Scale, 5th edition (WISC-V) [30]. Both methods represent a standardized developmental battery of neurocognitive tests. The test procedure takes about 90 min, and a well-trained psychologist presented tasks to the children. Both tests provide a general IQ-score (*M* = 100, *SD* = 15). Previous research has demonstrated moderate to high correlations between the IDS and the WISC-IV which shares substantial structural similarity with the WISC-V [35], supporting cautious interpretability of cognitive changes across time points.

### Child internalizing symptoms

Children’s internalizing symptoms were assessed using the German version of the Diagnostic System for Psychiatric Disorders according to the International Statistical Classification of Diseases and Related Health Problems 10th Revision (ICD-10) and the Diagnostic and Statistical Manual of Mental Disorders, 4th Edition (DSM-IV) for children and adolescents (T1: DISYPS-II; T2: DISYPS-III) [31, 32]. DISYPS III is an updated version of DISYPS II, and both versions are considered comparable [32]. At primary school-age and in early adolescence, mothers rated children’s disorder specific symptoms for anxiety and depression during the previous six months on 4-point-Likert scales, with *0 = not at all*, *1 = a little bit*, *2 = to a large extent*, and *3 = particularly*. Thus, all subscales yield in a total score (mean) with a theoretical range of 0.00 to 3.00. In early adolescence, T2 self-reports of participating children were also available. For this study, two subscales of DISYPS-II (T1) and DISYPS-III (T2) were used: Anxiety (44 items, e.g., “My child shows single intense anxiety states that develop within a few minutes”/“I am suddenly overcome by very strong fear, that develops in a few minutes”) and depression (29 items, e.g., “My child seems sad most of the time; often appears close to tears”/“I am sad most of the time and often close to tears”).

### Child HRQoL

Children’s quality of life was measured by the German version of the Pediatric Cardiac Quality of Life Inventory at T1 and T2 [33]. The inventory assesses the disease specific, pediatric HRQoL of children with CHD. In primary school-age and adolescence, mothers rated their children’s HRQoL on 26 items with a 5-point Likert-scale: *1 = always*,* 2 = often*,* 3 = sometimes*,* 4 = occasionally*, and *5 = never*, resulting in a total score of HRQoL (raw mean sum score). Raw mean sum scores were transformed into percentage scales with a theoretical range of 0–100%, with higher scores indicating higher HRQoL. In addition to mother-reports of HRQoL, children’s self-reports were available in early adolescence. All HRQoL questions started with “Because of my child’s heart problem…”/“Because of my heart problem…”.

### Maternal psychopathology

The German version of the Brief Symptom Inventory (BSI) was implemented to measure maternal psychopathology at T1 and T2 [34, 36]. This self-report questionnaire assesses clinically relevant psychopathological symptoms during the last seven days using 53 items on a 5-point Likert scale (*0 = not at all*,* 1 = a little bit*,* 2 = quite a bit*,* 3 = highly*,* 4 = very much*). The BSI covers nine symptom dimensions: Somatization, obsession-compulsion, interpersonal sensitivity, depression, anxiety, hostility, phobic anxiety, paranoid ideation and psychoticism. Examples for symptoms are “feeling fearful”, “faintness or dizziness”, or “feeling hopeless about the future”. For this study, the Global Severity Index (GSI) was used by transforming mean raw values into standardized T-scores according to norm tables (*M* = 50, *SD* = 10).

### Potential covariates

Child age and sex as well as families’ socio-economic status (SES) were examined as potential covariates. SES was calculated using data from the first time of measurement by conducting a sum index of families’ monthly net income in Euros (six levels: [1] < 1.000€ [2], 1.000€ – 2.000€ [3], 2.000€ – 3.000€ [4], 3.000€ – 4.000€ [5], 4.000€ − 5.000€ [6], > 5.000€), maternal and paternal secondary education level in years (for levels: [1] < 9y [2], 9y [3], 10-12y [4], 13y), and maternal and paternal migration background (mother or father immigrated ore moved into Germany; two levels: [0] yes [1], no), yielding a theoretical range of 3 to 16 [15].

### Statistical analyses

Outliers were analyzed using the outlier detection method based on Chebyshev’s theorem [37] with 95% of values varying ± 4.5 SD around the mean. Descriptive statistics were conducted and normal distribution was analyzed using Shapiro-Wilk tests. For examining the effects of VSD surgery characteristics and the hospital stay on mother-reported child psychosocial adjustment and maternal psychopathology from primary school-age to early adolescence, repeated measure analyses of covariance (rmANCOVAs) were conducted. Child neurodevelopment and psychosocial adjustment (internalizing symptoms and HRQoL) or maternal psychopathology at T1 and T2 were used as within-group factor, and characteristics of the VSD surgery and hospital stay were used as covariates. When rmANCOVA indicated a significant main effect of the covariate or significant interaction effect (time x covariate), post-hoc tests using Pearson’s correlation (*r*) between child neurodevelopment, psychosocial adjustment or maternal psychopathology and the covariate were conducted and coefficients were interpreted as > 0.10 small, > 0.30 medium, and > 0.50 large [38]. Effects of time were not reported as changes over time in child developmental outcomes and maternal psychopathology were not of interest in this study and have already been published elsewhere [14, 16]. Multiple linear regression analyses were performed to investigate effects of VSD surgery characteristics and the hospital stay (predictors) on self-reported child psychosocial adjustment (mental health, HRQoL) in adolescence (T2). All analyses were performed using IBM SPSS statistics (version 28, IBM Corporation, Armonk, NY, USA). Analyses were done two-tailed and the level of significance was defined as *p* < .05. Considering the small sample size, results with a level of significance *p* < .10 were reported as marginal significant. Partial eta-squared values (*η*^*2*^_*p*_) were reported as measures for effect sizes in rmANCOVAs with 0.01 ≤ *η*^*2*^_*p*_ ≥ 0.05 representing small, 0.06 ≤ *η*^*2*^_*p*_ ≥ 0.13 medium, and *η*^*2*^_*p*_ > 0.13 large effects [38].

## Results

### Preliminary analyses

Descriptive statistics on characteristics of VSD surgery and hospital stay are presented in Table 2. All measures (independent and dependent measures) were within a 4.5 SD range around the mean, following, no cases were excluded. However, one case had to be excluded due to a more complex medical history compared to the other participants. Potential confounding effects of child sex, age, and families’ socioeconomic status on dependent variables (child internalizing symptoms, HRQoL, and maternal psychopathology) were tested. Child sex and age showed no significant relations to dependent variables. As families’ socioeconomic status was positively related to child neurodevelopment (IQ), it was controlled in all analyses on child IQ.

**Table 2:** Descriptive statistics and intercorrelations of VSD surgery-related variables

Variable	*N*	*M (SD)*	*Min*	*Max*	*2.*	*3.*	*4.*	*5.*	*6.*
*Characteristics of surgery and hospital stay*									
1. Age at surgery (months)	23	12.78 (10.38)	1.38	33.18	− 0.27	− 0.20	− 0.29	0.43*	0.25
2. Duration of surgery (min)	23	230.48 (38.37)	175	355		0.90***	− 0.18	− 0.02	− 0.40^+^
3. Cardiopulmonary bypass time (min)	23	123.91 (26.36)	86	202			− 0.10	0.03	− 0.46
4. Post-surgical hospital stay (nights)	23	7.87 (3.22)	5	14				0.00	− 0.13
*Characteristics of the scar in adolescence*									
5. Scar length (cm)^a^	22	7.66 (1.29)	4.15	10.06					0.44*
6. Scar width (cm)^a^	22	0.44 (0.42)	0.01	1.78					

1 case had to be excluded in advance (outlier in terms of medical history)

^a^Measured at T2, values were relativized at the child's height to control for confounding effects

^+^p < .10, *p < .05, ***p < .001

### Effects of surgery-related variables on child development and maternal psychopathology

**Fig. 2:**
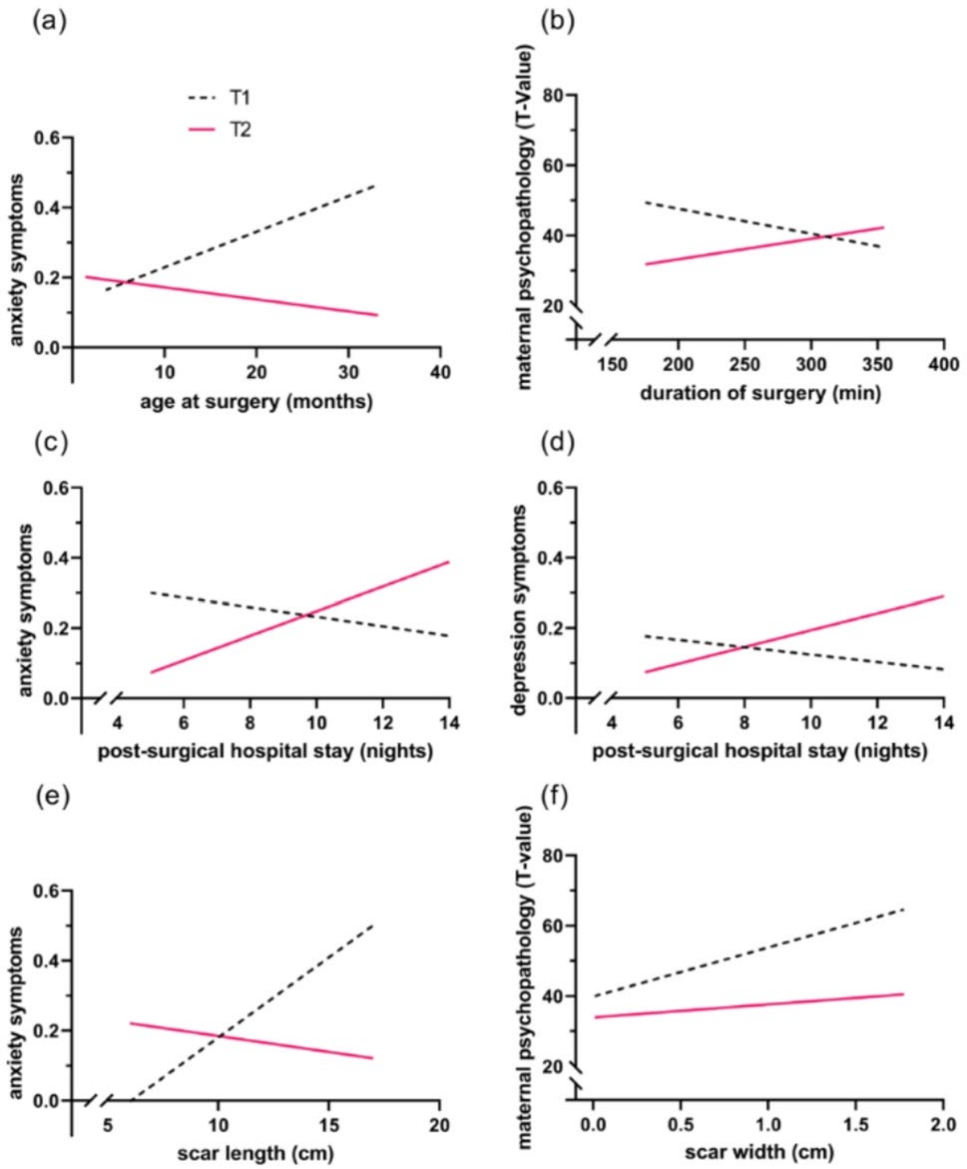
Significant time x covariate interactions (a) age at surgery (months) and child anxiety symptoms, (b) duration of surgery (min) and maternal psychopathology, (c) post-surgical hospital stay (nights) and child anxiety symptoms, (d) post-surgical hospital stay (nights) and child depression symptoms, (e) scar length (cm) and child anxiety symptoms, (f) scar width (cm) and maternal psychopathology

RmANCOVAs with T1 and T2 as repeated measures were used to analyze the effects of the characteristics of VSD surgery and the following hospital stay on mother-reported child psychosocial adjustment (IQ, anxiety, depression, and HRQoL) and maternal psychopathology. Results are depicted in Table 2 and significant time x covariate interactions are visualized in Fig. 2.

### Age at surgery

Analyzing the effects of child age at surgery, rmANCOVAs revealed no significant main or time x covariate interaction effects on child IQ, child depressive symptoms or maternal psychopathology.

*Anxiety*. Results revealed no significant main effect of the covariate child age at surgery. However, there was a significant time x covariate interaction (*F*(1, 15) = 5.73, *p* =.03, *η*^*2*^_*p*_ = 0.28). Post-hoc Pearson correlations showed a positive trend-significant association of the covariate and child anxiety symptoms at T1, and a non-significant negative one at T2 (*r*_*T1*_ = 0.40, *p* =.071); *r*_*T2*_ = − 0.13, *n.s.*; see Fig. 2a).

*HRQoL.* The covariate child age at surgery showed a significant main effect on child HRQoL (*F*(1, 16) = 5.26, *p* =.036, *η*^*2*^_*p*_ = 0.25). Post-hoc trend-significant Pearson correlations showed that a younger age at surgery was related to higher HRQoL both at primary school age and in adolescence (*r*_*T1*_ = − 0.50, *p* =.023; *r*_*T2*_ = − 0.41, *p* =.074). The time x covariate interaction was not significant.

### Duration of surgery

Taking the covariate duration of surgery into account, there were no significant main effects of the covariate and no significant time x covariate interactions on child IQ or internalizing symptoms (anxiety, depression).

*HRQoL*. Regarding child health-related quality of life, results showed a significant main effect of the covariate duration of surgery (*F*(1, 16) = 8.50, *p* =.010, *η*^*2*^_*p*_ = 0.35). Post-hoc analyses showed moderate significant associations between the covariate and child HRQoL of life at primary school-age and non-significant in adolescence, whereby children whose VSD surgery lasted longer had a higher quality of life (*r*_*T1*_ = 0.54, *p* =.023; *r*_*T2*_ = 0.32, *n.s.*). There was no significant time x covariate interaction.

*Maternal psychopathology*. Results of the rmANCOVA revealed no significant main effect of the covariate duration of surgery on maternal psychopathology. However, there was a significant time x covariate interaction (*F*(1, 20) = 5.08, *p* =.036, *η*^*2*^_*p*_ = 0.20). Post hoc analyses resulted in a non-significant negative relation of duration of surgery and maternal psychopathology at child primary school age (*r*_*T1*_ = − 0.23, *n.s.*), and in a non-significant positive association in child adolescence (*r*_*T2*_ = 0.26, *n.s.*). The time x covariate interaction is depicted in Fig. 2b.

### Cardiopulmonary bypass time

RmANCOVAs resulted in no significant main effects of cardiopulmonary bypass time and no significant time x covariate interactions on child IQ and internalizing symptoms (anxiety, depression) as well as on maternal psychopathology.

*HRQoL*. However, results revealed a significant main effect of the covariate on child HRQoL (F(1,16) = 6.23, *p* =.024, *η*^*2*^_*p*_ = 0.28). Post-hoc tests showed a significant moderate positive correlation between cardiopulmonary bypass time and children’s HRQoL at primary school-age (*r*_*T1*_ = 0.47, *p* =.034), and a small non-significant association in early adolescence (*r*_*T2*_ = 0.30, *n.s.*).

### Post-surgical hospital stay

The covariate post-surgical hospital stay showed no significant main effects or time x covariate interactions on child HRQoL or maternal psychopathology.

*IQ*. The covariate post-surgical hospital stay showed a marginal significant main effect on child neurodevelopment (IQ; *F*(1, 15) = 3.32, *p* =.088, *η*^*2*^_*p*_ = 0.18). At both measurement points, a longer post-surgical hospital stay was non-significantly related to lower IQ-scores with moderate effect sizes (partial correlations corrected for SES: *r*_*T1*_ = − 0.40, *n.s.*; *r*_*T2*_ = − 0.40, *n.s.*). There was no significant time x covariate interaction.

*Anxiety*. Regarding child anxiety symptoms, rmANCOVA revealed no significant main effect of the covariate. However, a significant time x covariate interaction was detected (*F*(1, 15) = 5.59, *p* =.032, *η*^*2*^_*p*_ = 0.27). Post-hoc analyses showed a non-significant negative correlation between the duration of the post-surgical hospital stay and child anxiety symptoms at primary school age (*r*_*T1*_ = − 0.17, *n.s.*), and a moderate trend-significant positive association in adolescence (*r*_*T2*_ = 0.41, *p* =.083). Results are depicted in Fig. 2c.

*Depression*. No significant main effect of post-surgical hospital stay on child depressive symptoms was detected. Results showed a highly significant time x covariate interaction (*F*(1, 16) = 15.84, *p* =.001, *η*^*2*^_*p*_ = 0.48), with post-hoc tests revealing a non-significant negative relation of post-surgical hospital stay and child depressive symptoms at T1 (*r*_*T1*_ = − 0.18, *n.s.*), and a moderate positive non-significant correlation at T2 (*r*_*T2*_ = 0.36, *n.s.*). For an illustration see Fig. 2d.

### Scar length

There were no significant main effects of the covariate length of the scar and no significant time x covariate interactions on child neurodevelopment (IQ), child depression or maternal psychopathology.

*Anxiety*. RmANCOVA showed no significant main effect of the covariate scar length on child anxiety symptoms. However, a marginal significant time x covariate interaction was found (*F*(1, 15) = 3.32, *p* =.088, *η*^*2*^_*p*_ = 0.18). Non-significant Pearson correlations as post-hoc analyses yielded in a positive relation of scar length and anxiety symptoms at primary school age (*r*_*T1*_ = 0.26, *n.s.*), and a small negative association in adolescence (*r*_*T2*_ = − 0.18, *n.s.*); see Fig. 2e.

*HRQoL*. Considering the length of the scar as a covariate, a marginal significant main effect of the covariate was found (F(1, 16) = 3.12, *p* =.096, *η*^*2*^_*p*_ = 0.17. Post-hoc Pearson correlations showed negative associations between the length of the scar and children’s HRQoL, with significance at primary school-age and non-significance in early adolescence (*r*_*T1*_ = − 0.43, *p* =.053; *r*_*T2*_ = − 0.25, *n.s.*).

### Scar width

Results of rmANCOVA revealed no significant main effects of the covariate and no significant time x covariate interactions on child IQ or anxiety symptoms.

*Depression*. Considering scar width as a covariate, there was a significant main effect of the covariate on child depressive symptoms (*F*(1, 16) = 5.76, *p* =.029, *η*^*2*^_*p*_ = 0.27). Pearson correlations revealed moderate associations of higher scar width and higher depressive symptoms, with significance at T1 and without significance at T2 (*r*_*T1*_ = 0.50, *p* =.022; *r*_*T2*_ = 0.34, *n.s.*). The time x covariate interaction was not significant.

*HRQoL*. RmANCOVA yielded in a main effect of the covariate on child HRQoL by trend (*F*(1, 16) = 3.59, *p* =.076, *η*^*2*^_*p*_ = 0.18). Pearson correlations showed negative relations between scar width and HRQoL that reached trend-significance at T1 and were non- significant at T2 (*r*_*T1*_ = − 0.39, *p* =.082; *r*_*T2*_ = − 0.19, *n.s.*). There was no significant time x covariate interaction.

*Maternal psychopathology*. There was a marginal significant main effect of the covariate on maternal psychopathology (*F*(1, 19) = 4.09, *p* =.058, *η*^*2*^_*p*_ = 0.18). In addition, results showed a marginal significant time x covariate interaction (*F*(1, 19) = 3.09, *p* =.095, *η*^*2*^_*p*_ = 0.14). Post-hoc Pearson correlations yielded in a significant moderate positive relation of child surgical scar width and maternal psychopathology at T1, and a non-significant small positive association at T2 (*r*_*T1*_ = 0.48, *p* =.026; *r*_*T2*_ = 0.18, *n.s.*; Fig. 2f).

**Table 3:** Repeated-measures ANCOVA-tested mean differences in child psychosocial adjustment (mother-reports) and maternal psychopathology (self-reports) considering surgery-related variables as covariates

	T1		T2			Covariate			Interaction time x covariate		
	*M*	*SD*	*M*	*SD*	Difference: T1 – T2 (95% CI)	*F*	*p*	η^2^_*p*_	*F*	*p*	η^2^_*p*_
*Child*						Age at surgery (months)			Time x age at surgery (months)		
IQ^a^	97.50	11.54	100.67	9.85	−3.17 (−6.52 to 0.18)	1.09	0.312	0.07	2.08	0.170	0.12
Anxiety	0.28	0.28	0.18	0.28	0.10 (−0.05 to 2.5)	0.33	0.575	0.02	5.73	0.030	0.28
Depression	0.16	0.20	0.15	0.22	0.01 (−0.08 to 0.10)	1.11	0.307	0.07	0.80	0.348	0.05
HRQoL	56.65	11.95	72.94	6.63	−7.30 (−12.58 to −2.02)	5.26	0.036	0.25	1.36	0.261	0.08
*Mother*											
Psychopathology	45.36	12.08	34.86	8.85	10.5 (5.52 to 15.48)	0.37	0.549	0.02	1.86	0.187	0.09
*Child*						Duration of surgery (min)			Time x duration of surgery (min)		
IQ^a^	97.50	11.54	100.67	9.85	−3.17 (−6.52 to 0.18)	1.55	0.232	0.09	0.52	0.480	0.03
Anxiety	0.28	0.28	0.18	0.28	0.10 (−0.05 to 2.5)	1.20	0.178	0.12	0.96	0.343	0.06
Depression	0.16	0.20	0.15	0.22	0.01 (−0.08 to 0.10)	0.94	0.346	0.06	0.06	0.811	0.00
HRQoL	56.65	11.95	72.94	6.63	−7.30 (−12.58 to −2.02)	8.50	0.010	0.35	3.01	0.102	0.16
*Mother*											
Psychopathology	45.36	12.08	34.86	8.85	10.5 (5.52 to 15.48)	0.01	0.920	0.00	5.08	0.036	0.20
*Child*						Cardiopulmonary bypass time (min)			Time x cardiopulmonary bypass time (min)		
IQ^a^	97.50	11.54	100.67	9.85	−3.17 (−6.73 to 0.38)	1.75	0.206	0.10	0.10	0.762	0.01
Anxiety	0.28	0.28	0.18	0.28	0.101 (−0.07 to 0.27)	1.18	0.294	0.07	1.00	0.332	0.06
Depression	0.16	0.20	0.15	0.22	0.01 (−0.07 to 0.10)	0.34	0.955	0.00	0.00	0.996	0.00
HRQoL	56.65	11.95	72.94	6.63	−7.30 (−12.37 to −2.23)	6.23	0.024	0.28	2.77	0.115	0.15
*Mother*											
Psychopathology	45.36	12.08	34.86	8.85	10.50 (5.63 to 15.37)	1.31	0.266	0.06	2.90	0.104	0.13
*Child*						Post-surgical hospital stay (nights)			Time x post-surgical hospital stay (nights)		
IQ^a^	97.50	11.54	100.67	9.85	−3.17 (−6.52 to 0.18)	3.32	0.088	0.18	0.00	0.989	0.00
Anxiety	0.28	0.28	0.18	0.28	0.10 (−0.05 to 2.5)	0.79	0.388	0.27	5.59	0.032	0.27
Depression	0.16	0.20	0.15	0.22	0.01 (−0.08 to 0.10)	0.44	0.517	0.03	15.84	0.001	0.48
HRQoL	56.65	11.95	72.94	6.63	−7.30 (−12.58 to −2.02)	0.23	0.641	0.01	1.18	0.294	0.07
*Mother*											
Psychopathology	45.36	12.08	34.86	8.85	10.5 (5.52 to 15.48)	0.00	0.972	0.00	0.00	0.973	0.00
*Child*						Scar length (cm)^b^			Time x scar length (cm)^b^		
IQ^a^	97.50	11.54	100.67	9.85	−3.17 (−6.52 to 0.18)	0.34	0.568	0.02	0.61	0.448	0.04
Anxiety	0.28	0.28	0.18	0.28	0.10 (−0.05 to 2.5)	0.02	0.898	0.00	3.32	0.088	0.18
Depression	0.16	0.20	0.15	0.22	0.01 (−0.08 to 0.10)	0.50	0.490	0.03	1.31	0.270	0.08
HRQoL	56.65	11.95	72.94	6.63	−7.30 (−12.58 to −2.02)	3.12	0.096	0.16	2.09	0.167	0.12
*Mother*											
Psychopathology	45.36	12.08	34.86	8.85	10.5 (5.52 to 15.48)	0.26	0.614	0.01	0.74	0.399	0.04
*Child*						Scar width (cm)^b^			Time x scar width (cm)^b^		
IQ^a^	97.50	11.54	100.67	9.85	−3.17 (−6.52 to 0.18)	0.00	0.958	0.00	0.19	0.666	0.01
Anxiety	0.28	0.28	0.18	0.28	0.10 (−0.05 to 2.5)	1.54	0.234	0.09	1.76	0.205	0.11
Depression	0.16	0.20	0.15	0.22	0.01 (−0.08 to 0.10)	5.76	0.029	0.27	1.03	0.326	0.06
HRQoL	56.65	11.95	72.94	6.63	−7.30 (−12.58 to −2.02)	3.59	0.076	0.18	2.98	0.104	0.16
*Mother*											
Psychopathology	45.36	12.08	34.86	8.85	10.5 (5.52 to 15.48)	4.09	0.058	0.18	3.09	0.095	0.14

^a^Controlled for socio-economic status: Additive combination of parental migration background, education level and the family income (theoretical range 3–16) [15]. ^b^Measured at T2, values were relativized at the child's height to control for confounding effects

IQ: T1, Intelligence and Development Scales, IDS-III [30]; T2, Wechsler Intelligence Scale for Children – Fifth edition, WISC-V [31]; child anxiety and depression: T1, DYSIPS-II [32], T2, DYSIPS-III [33]; HRQoL: PCQLI [34]; Maternal psychopathology: BSI [35]. Sample sizes: IQ *n* = 18; anxiety *n* = 17; depression *n* = 18; HRQoL *n* = 18; Maternal psychopathology *n* = 22

### Effects of surgery-related variables on adolescents’ self-reported psychosocial adjustment

To examine the effects of surgery-related variables on adolescents’ self-reported well-being (anxiety, depression, and HRQoL), multiple linear regressions models were conducted separately for each predictor (see Table 3). The number of nights of the post-surgical hospital stay significantly predicted a lower self-reported HRQoL of adolescents. Other characteristics of the VSD surgery, hospital stay and surgical scar had no significant effect on adolescent’s self-reported psychosocial adjustment.

**Table 4:** Regression analyses of adolescents’ self-reported psychosocial adjustment

	*R* ^2^	*F*	*df*	*β*	*p*
Anxiety					
Age at surgery (months)	0.01	0.16	1, 18	0.09	0.695
Duration of surgery (min)	0.03	1.68	1, 18	− 0.29	0.212
Cardiopulmonary bypass time (min)	0.05	0.90	1, 18	− 0.22	0.357
Post-surgical hospital stay (nights)	0.05	0.91	1, 18	0.22	0.354
Scar length (cm)^a^	0.00	0.02	1, 17	− 0.04	0.884
Scar width (cm) ^a^	0.01	0.16	1, 17	− 0.10	0.698
Depression					
Age at surgery (months)	0.00	0.03	1, 17	0.04	0.863
Duration of surgery (min)	0.07	1.33	1, 17	− 0.27	0.265
Cardiopulmonary bypass time (min)	0.05	0.96	1, 17	− 0.23	0.342
Post-surgical hospital stay (nights)	0.10	1.94	1, 17	0.32	0.182
Scar length (cm) ^a^	0.00	0.00	1, 16	0.00	0.991
Scar width (cm) ^a^	0.01	0.10	1, 16	0.08	0.761
HRQoL					
Age at surgery (months)	0.01	0.14	1, 18	− 0.09	− 0.709
Duration of surgery (min)	0.03	0.57	1, 18	0.18	0.461
Cardiopulmonary bypass time (min)	0.01	0.09	1, 18	0.07	0.769
Post-surgical hospital stay (nights)	0.21	4.75	**1, 18**	− 0.46	0.043
Scar length (cm) ^a^	0.02	0.25	1, 17	− 0.12	0.621
Scar width (cm) ^a^	0.00	0.02	1, 17	0.03	0.904

^a^Measured at T2, values were relativized at the child's height to control for confounding effects. Adolescents’ self-reported anxiety and depression: T1, DYSIPS-II [32], T2, DYSIPS-III [33]; HRQoL: PCQLI [34]

## Discussion

The aim of this study was to investigate the long-term effects of VSD surgery, hospital stay and scar characteristics on neurodevelopment and psychosocial adjustment of children after early surgical VSD repair during childhood and adolescence. Surgery-related variables included age at surgery (in months), duration of surgery (in minutes), cardiopulmonary bypass time (in minutes), post-surgical hospital stay (in number of nights), and scar length and width (in centimeters) at T2. We expected that older age at surgery, longer duration of surgery or cardiopulmonary bypass time, and longer post-surgical hospital stay would be linked to higher child internalizing symptoms (anxiety and depression) and associated with lower IQ scores and lower HRQoL. In addition, we analyzed the potential effects of child surgery-related variables on maternal psychopathology.

*Age at surgery.* It is standard practice to attempt surgical repair at less than one year of age because earlier intervention reduces the time that patients require aggressive medical therapy to prevent heart failure and minimizes the time these patients are exposed to increased pulmonary pressures, which can lead to irreversible heart and lung damage [6]. Early closure of a VSD can also lead to earlier normalization of growth parameters comparable to non-affected children [39]. Besides physical developmental outcomes, in our cohort a younger age at surgery was also linked to better psychosocial adjustment and well-being: In examining the relation between child anxiety symptoms and the age at which surgery was performed, our cohort showed higher levels of anxiety during primary school years and an older age at surgery. However, this association diminished until early adolescence. Other studies investigating psychosocial adjustment of children after heart-surgery due to complex CHD reported also on higher internalizing symptoms in patients with an older age at surgery [40]. Furthermore, an older age at surgery was related to lower HRQoL both at primary school-age and during adolescence. However, other studies did not find any effects of the age at surgery on children’s QoL [41, 42]. In sum, our study identified an older age at surgical VSD correction as a risk factor for children’s psychosocial adjustment and well-being. Moreover, and consistent with other findings [43], we found no effects of children’s age at surgery on maternal psychopathology.

*Duration of surgery & cardiopulmonary bypass time.* Because duration of surgery and cardiopulmonary bypass time were closely related in our study, they are discussed together in the following section. Both time-based, surgery-related variables had no effects on child neurodevelopment (IQ) and internalizing symptoms. Thus, with a longer duration of surgery and a longer cardiopulmonary bypass time, the circulation seems to remain intact and does not lead to damage in the brain, which would lead to impairments in cognitive development. However, both variables showed a positive main effect on children’s HRQoL at both measurement points. As shown in our study, quality of life was significantly higher in children with longer VSD surgery and longer cardiopulmonary bypass time. However, a longer duration of the child’s VSD surgery was associated with maternal psychopathology but this effect only became significant at T2. In line with this, Landolt and colleagues (2008) [44] also found that a longer duration of surgery and post-surgical hospital stay had a negative impact on parent-related, but not child-related quality of life. Moreover, others found no influence of the duration of heart-surgery on surgery-related stress disorder symptoms in parents of children who underwent pediatric cardiac surgery in the short-term (after 6 months) [45]. Our data extend this finding as we found no effects on maternal psychopathology at child primary school-age. The negative effect of a longer duration of surgery on maternal psychopathology only seems to come into play in child adolescence.

*Post-surgical hospital stay.* Prolonged length of stay after the heart surgery is often seen as a parameter for the severity of the clinical illness and is known to be associated with poor developmental outcomes [2]. Children with a longer post-surgical stay after early CHD surgery were reported to have lower scores on tests of cognitive outcomes in toddlerhood and primary school-age [23, 46]. Our results also suggested a marginal significant but large effect on children’s neurodevelopment. Although the IQ-scores of participating children varied in a normal range, a longer post-surgical hospital stay was related to lower IQ-scores both at primary school-age and in early adolescence. The specific mechanisms by which a longer duration of post-surgical hospital stay might predict poorer cognitive developmental outcomes after pediatric CHD surgery are uncertain [23]. A recent systematic review and meta-analysis suggests that cognitive developmental outcomes in CHD patients are largely determined by alterations in brain development or lesions that occur before pediatric cardiac surgery, so prenatal and postnatal processes may also play a role, leading to increased vulnerability to additional brain damage [2]. Moreover, patients with CHD are more likely at risk of developing psychopathologies with an approximately life time prevalence of 21,8% [47]. Furthermore, the level of psychopathological symptoms seem to be influenced by the duration of post-surgical hospital stay after surgical VSD correction reported by others [24]. In contrast, our cohort showed a small negative correlation with the length of post-surgical hospital stay and children’s internalizing symptoms (anxiety and depression) at primary school-age. However, at early adolescence, a longer post-surgical hospital stay was moderately associated with lower levels of internalizing symptoms. The adverse impact of post-surgical hospital stay appears to manifest primarily during adolescence. It is important to note that levels of internalizing symptoms (but not externalizing symptoms like ADHD and antisocial behavior) in our sample of children with surgically corrected VSD repair were elevated compared to those of a non-affected control group but varied still in a normal, sub-clinical range [15, 16]. Regarding the self-reported well-being of adolescents, a longer post-surgical hospital stay predicted lower HRQoL in our cohort at T2. Previous findings yielded mixed results, as some studies did find effects of the duration of post-surgical hospital stay on children’s HRQoL and some did not [18, 44, 48, 49]. To conclude, and in line with the recent meta-analysis of Huisenga and colleagues (2021), the duration of post-surgical hospital stay was identified as a crucial risk factor for child neurodevelopment, psychosocial adjustment and well-being after pediatric cardiac surgery. However, our data revealed no effects of the duration of post-surgical hospital stay on maternal psychopathology from child primary school-age to early adolescence. In contrast, others suggested that the post-surgical hospital stay may be stressful for parents and might lead to impairments in mental health, as they are confronted with insight of their mechanically ventilated child and potential alterations to their parental role [45, 50]. The different results may be due to the fact that our study examined the long-term consequences of early surgical VSD correction and not the psychosocial adaptation and development in the immediate postoperative period. Mothers therefore had plenty of time to adapt to the situation, which could have led to psychopathology scores varying in a normal range. In line with this, previous findings of our study demonstrated the possibility of regulation in the maternal stress system (biophysiological and psychological) of mothers with children who underwent early surgical VSD repair to the level of an unaffected control group, and no impairments in parenting behavior and mother-child relationship [14, 51].

*Surgical Scar.* The most prominent residual effect after cardiac surgery is the scar resulting from sternotomy, which is necessary to perform open-heart surgery and acts as a daily reminder of one’s heart defect. In adolescence, one’s physical appearance can become more relevant to confidence and self-esteem. Poor body image and embarrassment, especially among peers, can be significant psychological co-morbidities of sternotomy scars [26, 27]. Furthermore, median sternotomy scars can often result in discoloration, pruritus, chronic pain, and contracture, which can significantly reduce one’s overall quality of life. It is also significant that chronic pain can be particularly detrimental to quality of life in approximately 21% of patients [52]. In addition to influences of the surgical scar on body image, confidence and self-esteem, seeing the scar every day might be a continual reminder to the stressful event of heart surgery and the potentially traumatizing experience [53], both for the affected child and the mother. In our study, a longer scar was associated with higher anxiety symptoms at primary school-age; this finding seemed to be relativized in adolescence. Moreover, we found a marginal significant, negative relation of scar length and children’s mother-reported HRQoL at primary school age; this association also diminished until adolescence. However, the width of the scar seems to play an even more important role in the well-being of the child: Although no effect of scar width on children’s anxiety symptoms was found, results revealed large negative main effects on child depressive symptoms and HRQoL. Both at primary school-age and in adolescence, meaning stable over time, a wider surgical scar was linked to more child depressive symptoms and lower HRQoL. However, both associations tended to diminish as adolescents progress through this developmental phase. In line with this, Spijkerboer and colleagues (2010) reported a negative effect of a moderately or poorly healed scar on internalizing symptoms in 7–17 year olds after CHD surgery [19]. In addition, a marginal significant interaction effect regarding maternal psychopathology was found. At T1, a wider scar was associated with higher maternal psychopathology. This association tended to decrease in adolescence, suggesting that mothers may have adapted to their children’s circumstances. In line with this, previous results from our study also showed an adaptation from child primary school-age to adolescence of the altered biophysiological stress system in the mothers of children who underwent early surgical VSD repair [14]. To conclude, our study clearly shows that the characteristics of the remaining scar after an early surgical VSD repair play a long-term role in the psychosocial adjustment and well-being of affected children and their mothers. From a psychological perspective, this is why we recommend that pediatric cardiac surgeons try to make the scar as narrow, short and subtle as possible. Children who took part in the present study underwent surgical VSD repair between 2006 and 2012, so it can be assumed that the surgical technique is now more advanced than at the time of the participating children’s surgery. Research has already been conducted on less invasive surgical techniques and improved postoperative management [26, 54], yielding promising results for subtle scarring.

*Interrelations of surgery-related variables.* As shown by our results and reported by others, surgery-related variables are not independent of each other, but interrelated to varying degrees. Moreover, if surgery-related variables are identified as risk factors for child neurodevelopment, psychosocial adjustment and well-being, an accumulation of risk factors can occur leading to even worse child developmental outcomes. For example, a longer duration of surgery and especially a longer cardiopulmonary bypass time or a longer ischemic time, may also lead to a longer post-surgical hospital stay due to more complex CHD or complications during heart surgery, which in turn may require a longer recovery period. Furthermore, a younger age at surgery also carries higher risk for prolonged post-surgical hospital stay [55]. At the same time, a younger age surgery is associated with better scar results [56], which might be protective against impairments in psychosocial adjustment and well-being due to visible surgery scars.

### Limitations and future directions

Regarding statistical analyses, it is essential to address the methodology employed to evaluate the proposed hypotheses. To analyze the impact of one surgery-related variable on child development and maternal psychopathology, five rmANCOVAs were conducted separately for each outcome variable. The choice of data analysis approach was driven by the objective of maximizing test power in light of the limitations imposed by the relatively small sample size. In the context of multiple testing, the application of alpha-level corrections was considered, but ultimately not adopted, in light of the exploratory nature of the study and the relatively small sample size. The interpretation of the results must take effect size measures into account, and the constantly large effect sizes indicate the practical relevance of our findings. However, it must be acknowledged that samples of a limited size, such as the present one, can include individual cases that are extreme due to the influence of underlying medical complications, and thus exert a disproportionate effect on the results. While all values fell within a range of ± 4.5 SD, rendering them not outliers, the findings should be replicated in a larger sample to ensure the relative weighting of individual cases is accurately determined. Moreover, in instances where time x covariate interactions of the rmANCOVAs were identified as significant, only one potential direction of the interaction was subjected to post-hoc analysis. This entailed the calculation of correlations at both measurement time points, with the limitation that testing the second potential direction of the interaction, for instance, in relation to risk groups, was not feasible due to the restricted sample size. Another limiting factor is that our study only included biomedical, surgery-related variables to predict child neurodevelopment, psychosocial adjustment and well-being after early surgical VSD repair. However, emerging research is highlighting the combined influence of a variety of biomedical and psychosocial variables for a child’s experience in the long-term [57]. It is recommended that future studies include predictor variables of both areas and analyze potential mutual interactions in order to elucidate the child’s development and psychosocial adjustment best subsequent to early CHD surgery [28].

## Conclusion

Surgery-related variables of an isolated VSD repair in the first three years of life have significant long-term consequences for children’s neurodevelopment and psychosocial adjustment from primary school-age to early adolescence. In sum, especially the psychosocial adjustment and not child neurodevelopment was affected by surgery-related variables. Surprisingly, surgical time-based variables (duration of surgery, cardiopulmonary bypass time) showed less negative impact on child development than postoperative variables, such as the length of post-surgical hospital stay and scar characteristics. The length of the post-surgical hospital stay was identified as a highly relevant risk factor for later child development, indicating that special support, e.g., targeted promotion of child cognitive development and psychological support in case of stress or psychopathological symptoms should be offered to families affected by long hospital stays after surgery. These results suggest the attempt to reduce post-surgical hospital stay, as far as medically possible. This is already a body of research in pediatric cardiology departments with ventilation time, age at surgery and severity of CHD to be significant parameters for prolonged post-surgical hospital stay [58, 59]. The adverse long-term effects of a lengthy and wide scar on the psychosocial adjustment of children emphasize the significance of investing effort into the management of subtle scarring during pediatric cardiac surgery and subsequent care. In contrast to pre-operative variables (e.g., severity of CHD, child health status), many intra-operative techniques and shaping the surgical scar are modifiable, which may be altered to improve long-term child psychosocial adjustment and well-being [60]. To conclude, pre-, peri- and post-operative management plays a major role not only for somatic correction, but also for long-term psychosocial adjustment, both for affected children and their mothers.

## Data Availability

The data presented in this study are available on reasonable request from the corresponding author. The data are not publicly available due to privacy restrictions and the data protection law in Germany.

## Abbreviations

CHD: Congenital heart disease
HRQoL: Health-related quality of life
VSD: Ventricular septal defect
